# Efficiently photo-charging lithium-ion battery by perovskite solar cell

**DOI:** 10.1038/ncomms9103

**Published:** 2015-08-27

**Authors:** Jiantie Xu, Yonghua Chen, Liming Dai

**Affiliations:** 1 Center of Advanced Science and Engineering for Carbon (Case4Carbon), Department of Macromolecular Science and Engineering, Case Western Reserve University, Cleveland, Ohio 44106, USA

## Abstract

Electric vehicles using lithium-ion battery pack(s) for propulsion have recently attracted a great deal of interest. The large-scale practical application of battery electric vehicles may not be realized unless lithium-ion batteries with self-charging suppliers will be developed. Solar cells offer an attractive option for directly photo-charging lithium-ion batteries. Here we demonstrate the use of perovskite solar cell packs with four single CH_3_NH_3_PbI_3_ based solar cells connected in series for directly photo-charging lithium-ion batteries assembled with a LiFePO_4_ cathode and a Li_4_Ti_5_O_12_ anode. Our device shows a high overall photo-electric conversion and storage efficiency of 7.80% and excellent cycling stability, which outperforms other reported lithium-ion batteries, lithium–air batteries, flow batteries and super-capacitors integrated with a photo-charging component. The newly developed self-chargeable units based on integrated perovskite solar cells and lithium-ion batteries hold promise for various potential applications.

## Abstract (web summary)

Photo-charged battery devices are an attractive technology but suffer from low photo-electric storage conversion efficiency and poor cycling stability. Here, the authors demonstrate the use of perovskite solar cells in conjunction with a lithium ion battery which displays excellent properties.

## Graphical abstract

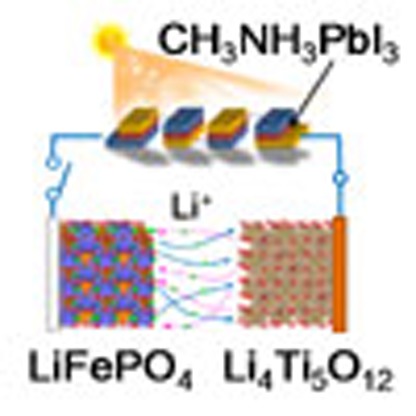

The importance of developing new types of energy conversion and storage systems is evident by the ever-increasing human reliance on energy-based appliances, the rapidly diminishing fossil fuels and the continuously growing environmental concerns. The global energy consumption by automobiles, along with the associated green-house-gas emission, has been accelerating at an alarming rate due to the rapid increase in world population and economic expansion worldwide. Unlike traditional vehicles relying heavily on the fossil fuels, electric vehicles or plug-in hybrid electric vehicles hold great promise to solve current energy and environmental challenges. With recent advance in the development of eco-friendly rechargeable lithium-ion batteries (LIBs) of higher power and energy densities^1^, battery electric vehicles (BEVs) using LIB pack(s) for propulsion have attracted a great deal of interest^2^. Of particular interest, Tesla has recently demonstrated the use of a battery pack consisting of 7,000 cylindrical LIB cells (each with 18 mm in diameter × 65 mm in length) connected in series and parallel to power electric vehicles (http://www.cadex.com/en/batteries/safety-of-lithium-ion-batteries), opening a new era of transportation with BEVs^3^. Although the excellent electrochemical performance of LIBs, including their high specific capacity, power and energy density, and rate capability, could ensure a long cruise duration, short charging time and low total cost of a BEV, implementing the BEV technology in our daily life is still a big challenge as it requires a cross-country network of BEV-charging stations. This will not only cause inflexibilities to travel plans but also inevitably increase the total cost of the BEV technology. Therefore, the large-scale practical application of BEVs cannot be realized unless LIBs with self-charging suppliers will be achieved.

The solar cell technology that can generate electricity from the sunlight^4^,^5^, could offer a viable approach to ‘self-charging' of LIBs wherever needed. With the free and abundant sunlight that provides about 10,000 times more energy to the Earth than we consume, solar cells can ensure sustainable access to electrical power for charging LIBs anywhere around the world with no air pollution, hazardous waste or noise, and they requires little upkeep. If solar cells can be used for directly photo-charging LIBs, therefore, the BEV technology could be marketed for large-scale applications even without the presence of a cross-country network of charging stations. Although conceptually promising, photo-charging of batteries, including LIBs, with solar cells has been much less discussed than photo-charging super-capacitors and is far from practical so far (Supplementary Table 1)^6^,^7^,^8^,^9^,^10^,^11^,^12^,^13^,^14^,^15^,^16^,^17^,^18^,^19^,^20^,^21^,^22^,^23^. This is because all the limited number of the photo-charging LIB devices reported to date exhibited a very low overall photo-electric storage-conversion efficiency and poor cycling stability (Supplementary Table 1).

One of the critical issues needs to be addressed for efficient photo-charging of LIBs is to enhance the usually low current density and power-conversion efficiency associated with most current solar cells, particularly polymer solar cells. In this regard, the recent availability of high-performance perovskite solar cells (PSCs)^5^,^24^,^25^,^26^,^27^,^28^,^29^,^30^,^31^ could not only facilitate the development of highly efficient (up to ∼20%) and low cost solar cells for practical applications but also allow for the integration of PSCs into various energy systems. Herein, we report the first use of PSC packs with four single CH_3_NH_3_PbI_3_ PSCs connected in series for directly photo-charging LIBs assembled with a LiFePO_4_ cathode and a Li_4_Ti_5_O_12_ anode. The fabricated PSC–LIB units were shown to possess a high overall photo-electric conversion and storage efficiency of 7.80% and excellent cycling stability—outperformed all other reported LIBs, lithium–air batteries, flow batteries and super-capacitors integrated with a photo-charging component. This work clearly indicates that the PSC–LIB units developed in this study hold great promise for potential applications as self-chargeable batteries to power the BEVs and various portable electronics.

## Results

### Working mechanism of a PSCs–LIB unit

**Figure 1:**
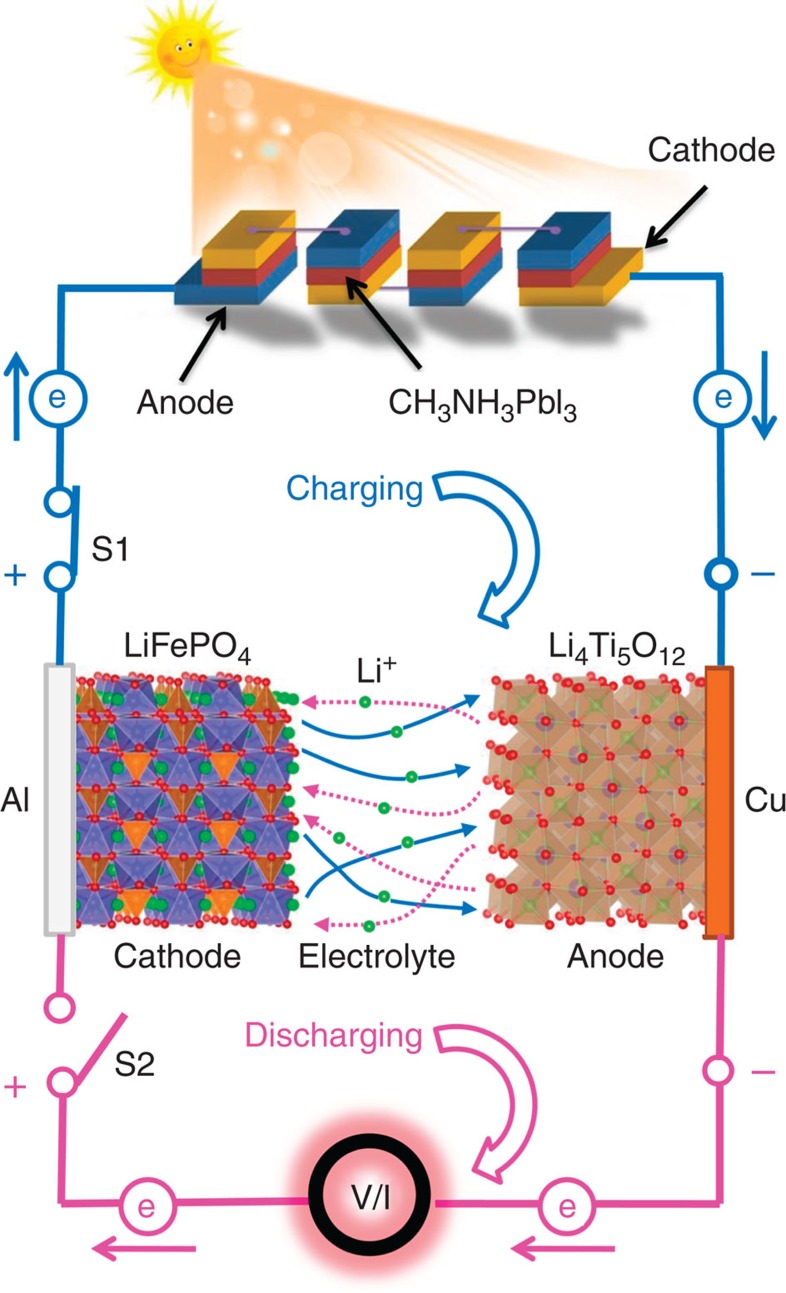
Photo-charging a LIB by PSCs. Schematic diagram of the fabricated system of PSC–LIB.

Figure 1 schematically shows the fabricated system for photo-charging a LIB by PSCs connected in series. As shown in Fig. 1, the photogenerated free holes and electrons within the PSCs flow into the cathode and anode of the LIB, respectively. This current flow from the PSC to the close circuited LIB (top, Fig. 1) leads to charging the LIB through oxidative extraction of lithium ions from the cathode (for example, olive structure LiFePO_4_), followed by reductive insertion onto the anode (for example, spinel structure Li_4_Ti_5_O_12_). The energy (charge) stored during the charging process can be released to external loading (bottom, Fig. 1) by turning S1 off and S2 on, which is accompanied by a simultaneous back flow of lithium ions from the anode to cathode.

### Fabrication and performance of PSCs

To construct the PSCs–LIB system shown in Fig. 1, we first fabricated PSCs with a device structure of ITO (indium tin oxide)/poly(3,4-ethylenedioxythiophene):poly(styrenesulfonate) (PEDOT:PSS) (40 nm)/CH_3_NH_3_PbI_3_ (perovskite) (375 nm)/[6,6]-phenyl-C61-butyric acid methyl ester (PC61BM) (100 nm)/Ca (20 nm)/Al (100 nm). Supplementary Fig. 1 schematically shows structures of the perovskite material and solar cell device while their characterization and fabrication details can be found in Supplementary Note 1 and Supplementary Methods. The CH_3_NH_3_PbI_3_ perovskite layer was prepared according to our previously reported layer-by-layer process^29^. Briefly, we first thermally evaporated a thin (125 nm) layer of lead iodide (PbI_2_) onto a PEDOT:PSS layer pre-coated on an ITO/glass substrate (Supplementary Fig. 1). Subsequently, we dipped the PbI_2_ into a solution of CH_3_NH_3_I in 2-propanol (10 mg ml^−1^) to transfer it into a layer of CH_3_NH_3_PbI_3_ perovskite. Similarly, a second PbI_2_ layer (125 nm) was thermally evaporated onto the newly formed CH_3_NH_3_PbI_3_ perovskite film, followed by dipping into the CH_3_NH_3_I solution to form the second layer of CH_3_NH_3_PbI_3_ perovskite intimately contacted with the underlying pre-formed CH_3_NH_3_PbI_3_ perovskite layer. The above process was repeated three times to form an interface-free uniform CH_3_NH_3_PbI_3_ perovskite layer of a desirable total thickness for outstanding photovoltaic performance required for photo-charging LIBs. The individual layer thickness of PbI_2_ was optimized at 125 nm as a thinner layer PbI_2_ (<125 nm) cannot ensure pinhole free, whereas a thicker PbI_2_ (>125 nm) cannot be totally transformed into CH_3_NH_3_PbI_3_ perovskite (Supplementary Fig. 2a). A typical scanning electron microscopy image for the resultant CH_3_NH_3_PbI_3_ perovskite film is given in Supplementary Fig. 2b, which shows a uniform, pinhole-free morphology. Supplementary Fig. 2c shows a typical X-ray diffraction pattern with peaks at 13.68°, 24.01°, 28.04° and 42.80° characteristic of the (110), (202), (220) and (330) diffractions, respectively, from the tetragonal CH_3_NH_3_PbI_3_ perovskite, indicating a complete transformation of the three-layered PbI_2_ with a total thickness of 375 nm into the CH_3_NH_3_PbI_3_ perovskite film. Further evidence for the complete conversion of the PbI_2_ layer into the CH_3_NH_3_PbI_3_ perovskite film came from the optical absorption spectrum given in Supplementary Fig. 2d, which shows a broad band with an onset at 770 nm characteristic of the CH_3_NH_3_PbI_3_ perovskite. The chemical composition of the CH_3_NH_3_PbI_3_ perovskite film was measured by X-ray photoelectron spectroscopy (XPS). Supplementary Fig. 3a shows an XPS survey spectrum while Supplementary Fig. 3b–e reproduce the high-resolution XPS spectra of C 1s, I 3d, Pb 4f and N 1s, respectively. Numerical data from the XPS measurements are listed in Supplementary Table 2. As expected, the atomic ratio of I/Pb was found to be 2.85, which is close to the stoichiometric value of 3.

**Figure 2:**
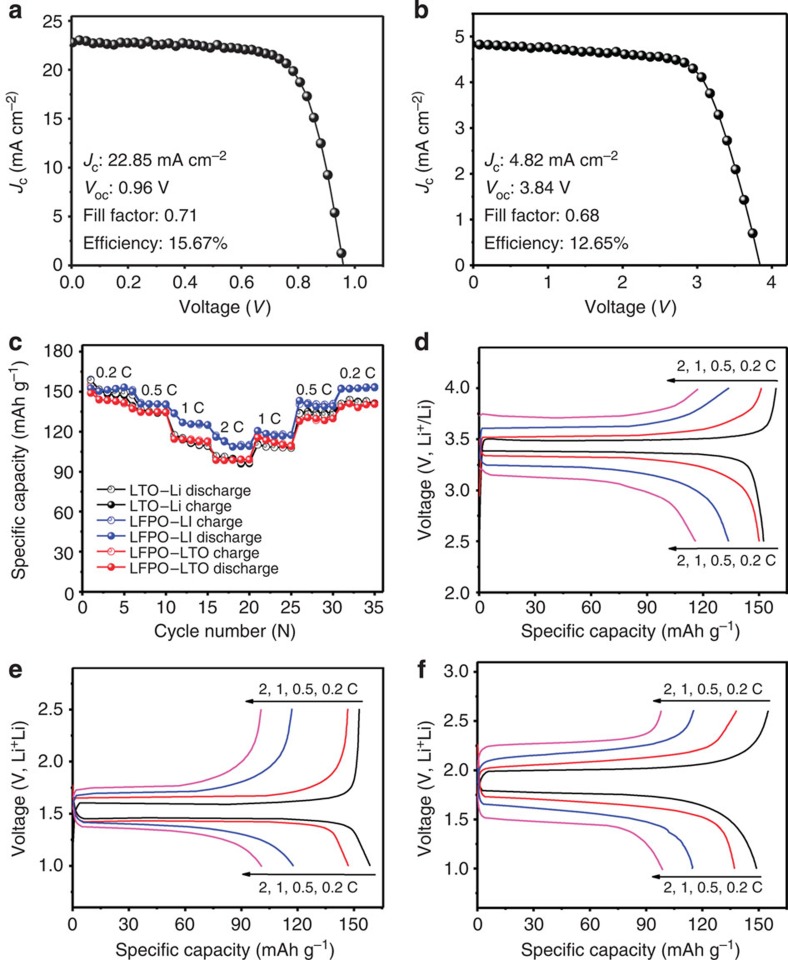
Performance of PSCs and LIBs. (**a**) *J*–*V* curve for single PSC. (**b**) A typical *J*–*V* curve for the connected PSCs unit with four single PSCs connected in series. (**c**) Rate capabilities of LFPO–Li, LTO–Li and LFPO–LTO cells measured at various C-rates (1 C=170 mA g^−1^) in the voltage ranges of 2.5–4.0, 1.0–3.0 and 1.0–2.6 V, respectively. (**d**–**f**) Typical charge–discharge curves of (**d**) LFPO–Li, (**e**) LTO–Li and (**f**) LFPO–LTO cells.

Before the system integration, we have further investigated the PSC performance. Figure 2a,b show typical current density–voltage (*J*–*V*) characteristics for a single PSC and four of them connected in series, respectively. For the single PSC, a short-circuit photocurrent density of 22.85 mA cm^−2^, open-circuit voltage of 0.96 V, fill factor of 0.71 and power-conversion efficiency (PCE; *η*_1_, Method calculation 1) of 15.67% were obtained. The short-circuit current density matched well with the current density of 21.93 mA cm^−2^ calculated from the incident photon-to-current efficiency (IPCE) spectrum (Supplementary Fig. 4). To ensure a sufficiently high operating voltage for directly photo-charging LIBs, we then fabricated a PSC pack by connecting four single cells in series (top, Fig. 1). The detailed *J*_c_, *V*_oc_, FF and *η*_1_ of these four single cells were shown in Supplementary Figs 5a and 6. As a result, we obtained a short-circuit photocurrent density of 4.82 mA cm^−2^ (per the total area of four PSCs), open-circuit voltage of 3.84 V, fill factor of 0.68 and PCE of 12.65% for the connected PSC unit (Fig. 2b). While the connected PSC unit exhibited sufficiently good performance with a high open-circuit voltage (3.84 V) for photo-charging LIBs, its long-term operation stability was tested, along with the corresponding single cells, to be superb even up to 720 h (in Ar atmosphere).

### Fabrication and electrochemical performance of LIBs

Owing to their high energy density, excellent thermal and chemical stability, long cycling life and superior safety, LiFePO_4_ (LFPO) and Li_4_Ti_5_O_12_ (LTO) have been widely regarded as promising cathode and anode materials, respectively, for the next generation of high-energy LIBs attractive for BEVs^1^,^32^,^33^. In this regard, we have fabricated a full LIB cell based on LiFePO_4_–1M LiFP_6_ in ethylene carbonate/dimethyl carbonate/diethyl carbonate (v/v/v: 1:1:1)–Li_4_Ti_5_O_12_ with a working voltage range of 1.0–2.6 V (Methods). The structure and morphology of the LFPO and LTO electrodes were confirmed by X-ray diffraction measurements (Supplementary Fig. 7a,b) and scanning electron microscopy images (Supplementary Fig. 7c,d). The electrochemical performance of a LFPO–Li or LTO–Li half cell (lithium foil as a counter and reference electrode) and a full cell of LFPO–LTO (with a LFPO/LTO mass ratio of ∼1.1) was given in Fig. 2c–f. Specifically, Fig. 2c shows the rate capability of the LFPO and LTO electrodes with respect to a full LFPO–LTO cell at the same C-rates. As can be seen, the LFPO and LTO electrodes exhibited initial charge/discharge capacities of 159.1/152.5 and 152.8/158.6 mAh g^−1^ at 0.2 C (1 C=170 mA g^−1^), and 151.2/150.1 and 139.4/147.5 mAh g^−1^ at 0.5 C, respectively. Figure 2d,e show typical charge–discharge profiles for the LFPO–Li and LTO–Li half cells, respectively, at various C-rates, showing the flat voltage profiles without significant over-potential at the C-rate ≤0.5 C. Similar flat discharge–charge profiles were observed for the LFPO–LTO cell. As shown in Fig. 2c,f, the LFPO–LTO cell exhibited a good cycling stability over a wide range of C-rates from 0.2 to 2 C with average charge/discharge capacities of 145.3/144.3 mAh g^−1^ at 0.2 C and 135.7/135.1 mAh g^−1^ at 0.5 C.

### Performance of PSCs–LIB device

**Figure 3:**
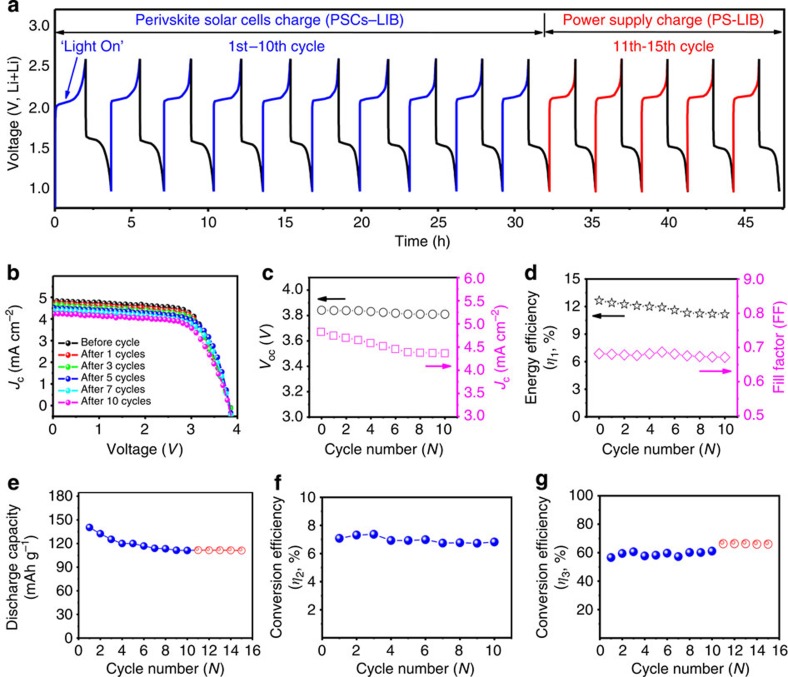
Performance of fabricated PSCs–LIB and PS-LIB. (**a**) Voltage–time (*V*–*t*) curves of the PSCs–LIB device (blue and black lines at the 1st–10th cycles: charged at 0.5 C using PSC and galvanostatically discharged at 0.5 C using power supply. Red and black lines at the 11th–15th cycles: both galvanostatically charged and discharged at 0.5 C using power supply). (**b**) *J*–*V* curves, (**c**) *V*_oc_ and *J*_c_, (**d**) fill factor (FF) and solar-to-electric PCE (*η*_1_) of the connected PSCs before and after various cycles. (**e**) Discharge capacity (Note, a slight variation was observed for the discharge duration at different cycle numbers), (**f**) overall photo-electric conversion efficiency of the PSCs–LIB device (*η*_2_) and (**g**) energy storage (conversion) efficiency (*η*_3_) of LIB, as a function of the cycle number.

Having carried out systematic performance evaluations for both the PSCs and LFPO/LTO-based LIBs, we used the PSC unit with four single PSCs connected in series to directly photo-charge a LFPO–LTO LIB at an current density of 0.5 C (0.085 mA g^−1^, 1 C=170 mA g^−1^), which matches well with the photogenerated current density from the PSC unit over the voltage range of 2.0–2.5 V (4.61–4.52 mA cm^−2^ and 0.087–0.085 mA g^−1^, Fig. 2b). Figure 3a shows photo-charge (blue lines) and galvanostatic discharge (black lines) curves of a LIB repeatedly photo-charged by the connected PSC unit (PSCs–LIB) for 10 cycles at 0.5 C between 1.0 and 2.6 V. To compare with the photo-charging, we also performed galvanostatic charge–discharge of the same LIB cell, using an automatic battery tester system (Land, China) as power supply (PS) after the completion of the 10 photo-charge/galvanostatic discharge cycles (PS-LIB, red lines in Fig. 3a). As shown in Fig. 3a, the PSCs–LIB and PS-LIB cells showed almost identical very stable discharge–charge curves over all the cycles, indicating excellent capabilities of the PSCs for photo-charging LIBs. Even after continued illumination under AM1.5G for 17.8 h over 10 repeated photo-charge and galvanostatic discharge cycles (Supplementary Fig. 8), the connected PSC unit showed only slight changes in all the photovoltaic characteristics, including *J*_c_, *V*_oc_ and FF, with a small decrease in PCE (*η*_1_) from 12.65 to 11.16% (Fig. 3b–d, Supplementary Table 3), corresponding to an average of 0.15% per cycle decay in *η*_1_. The above observed operation stability was also confirmed by the high *η*_1_ retention (∼93%) obtained for single (Supplementary Fig. 9a–c) and connected PSCs out of the photo-charging system (Fig. 1, Supplementary Fig. 9d–f) even after 720 h testing in an Ar-filled glove box without any encapsulation protection. As seen in Fig. 3e (blue dots), the fabricated PSCs–LIB cell delivered an initial reversible capacity of 140.4 mAh g^−1^ while maintained a reversible capacity of 111.6 mAh g^−1^ after 10 photo-charge and galvanostatic discharge cycles (that is, 79.49% initial capacity, ∼2.05% decay per cycle). This was followed by a highly reversible capacity of 111.1 mAh g^−1^ during the PS-LIB process up to the 15th galvanostatic charge–discharge cycle (Fig. 3e, red dots).

**Figure 4:**
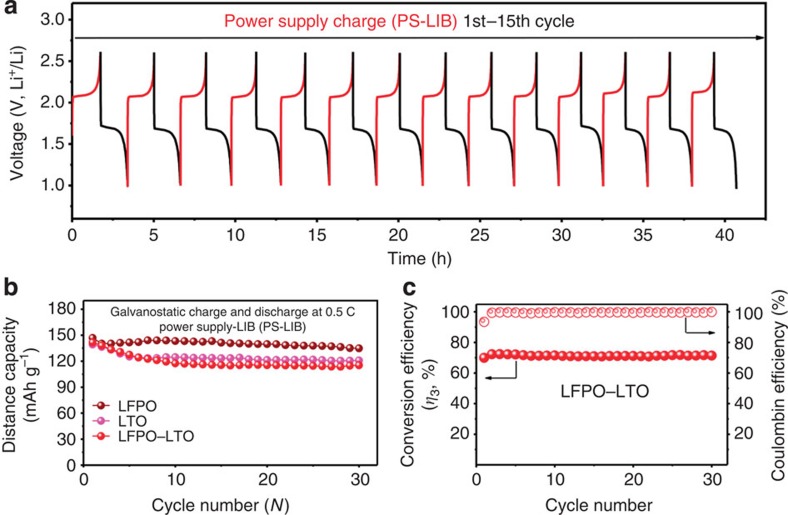
Cycling performance of the LTO, LFPO and LFPO–LTO. (**a**) *V*–*t* curves of a fresh LFPO–LTO cell measured at 0.5 C in the voltage range of 1.0–2.6 V for 15 cycles. (**b**) Cycling performance of LTO, LFPO and LFPO–LTO cells measured at 0.5 C in the voltage ranges of 1.0–3.0, 2.5–4.0 and 1.0–2.6 V, respectively, for 30 cycles. (**c**) Conversion efficiency (*η*_3_) and Coulombic efficiency of LFPO–LTO for 30 cycles.

In comparison, the galvanostatic charge–discharge cycles measured for fresh LFPO–Li, LTO–Li and LFPO–LTO cells at 0.5 C in the voltage ranges of 2.5–4.0, 1.0–3.0 and 1.0–2.6 V, respectively, for 30 cycles, using the automatic battery tester system as power supply (that is, PS), were given in Fig. 4. As expected, the fresh LFPO–LTO cell (Fig. 4a) using PS showed the similar charge–discharge *V*–*t* curves to that of (PSCs and PS)-LIB (Fig. 3a). As shown in Fig. 4b, the fresh cell delivered the initial, 11th, 15th and 30th reversible capacities of 141.6, 117.5, 115.5 and 115.2 mAh g^−1^, corresponding to an average capacity decay of 1.70% at the 10th cycle, 1.85% at the 15th cycle and 1.88% at the 30th cycle, during the galvanostatic charge and discharge cycles. As shown in Fig. 4b, the cycling stability of LFPO–LTO cell was significantly improved after initial few cycles due to the enhanced compatibility between the electrode and electrolyte. Along with the almost identical electrochemical behaviour (Fig. 4a), the close discharge capacity and average capacity decay observed for both the PS-LIB and PSCs–LIB indicate, once again, the excellent photo-charging capability of the PSCs and high cycling stability of the LFPO–LTO LIBs.

To gain insights into the power-conversion and storage efficiencies, we calculated the overall photo-electric conversion efficiency of the fabricated PSCs–LIB system (*η*_2_) by dividing the discharge energy of the LFP–LTO cell shown in Fig. 1 by the illumination energy (Method calculation 2). Figure 3f (blue dots) shows a rather stable *η*_2_ for the PSCs–LIB system during the 10 photo-charge and galvanostatic discharge cycles at 0.5 C, with a maximum *η*_2_ of 7.36% and an average *η*_2_ of 6.97% (Supplementary Table 4). Owing to a low potential polarization between the charge and discharge voltage plateaus at lower C-rates (cf. Fig. 2f), the PSCs–LIB exhibited an increased *η*_2_ with decreasing C-rate: 7.80% at 0.1 C, 7.35% at 0.25 C, 6.87% at 0.5 C, 6.47% at 0.75 C and 6.11% at 1 C (Supplementary Table 5). To our best knowledge, the overall 7.80% photo-electric conversion efficiency (*η*_2_) for the PSCs–LIB unit outperformed all other reported LIBs^7^, lithium–air batteries^20^, flow batteries^11^,^14^ and super-capacitors^10^,^19^,^23^ integrated with a photo-charging component, such as a solar cell (Supplementary Table 1). Furthermore, the energy storage efficiency (*η*_3_) of the LIB in the PSCs–LIB was calculated by *η*_2_/*η*_1_ (that is, Method calculation 3, blue dots in Fig. 3g) to be∼60% while *η*_3_ for the LIB in the PS-LIB was calculated to be ∼66% (Method calculation 4, red dots in Fig. 3g). The single fresh LFPO–LTO LIB showed a *η*_3_ of ∼71% and an average Coulombic efficiency of ∼99.5% (Fig. 4c). Clearly, therefore, the newly developed PSCs–LIB system possesses the highest *η*_1_, *η*_2_ and *η*_3_ among all reported photo-chargeable energy devices, including batteries and capacitors (Supplementary Table 1), holding great promise as a self-chargable power supplier for BEVs and many other optoelectronic systems.

## Discussion

As can be seen from above, the solar cell technology offers a viable option to self-charge LIBs for potentially marketing the BEV technology for large-scale applications. However, all the photo-charging LIB devices reported to date exhibited a very low overall photo-electric storage-conversion efficiency and poor cycling stability (Supplementary Table 1). We have, for the first time, demonstrated the use of PSC packs for directly photo-charging LIBs. The solar cell pack with four single CH_3_NH_3_PbI_3_ PSCs connected in series showed an outstanding solar-to-electric PCE of 12.65% (15.67% for the single cell), while the full LIB cell assembled with the LiFePO_4_ cathode and Li_4_Ti_5_O_12_ anode exhibited a high specific capacity and rate capability. The fabricated PSCs–LIB units were demonstrated to possess a high photo-electric conversion and storage efficiency of 7.80% and excellent cycling stability under continued illumination with AM1.5G for 17.8 h. For practical applications, repeatable and reliable fabrication of the PSCs–LIB units with good performance is another important issue needs to be addressed. Supplementary Figs 5b, 6 and 10 show a good repeatability for reliable fabrication of PSCs and integration of the resultant PSCs units for efficient photo-charging LIBs. In addition, an excellent discharge rate capability at various discharge rates was observed for the LFPO–LTO cell in the PSCs–LIB system (Supplementary Fig. 11). Both the photo-charged PSCs–LIB and the PS-LIB cells, gavanostatically charged and discharged at 0.5 C, exhibited a similar rate capability from 0.1 to 1 C (Supplementary Fig. 12), further indicating a highly stable PSCs–LIB system. To our best knowledge, the newly developed PSCs–LIB units outperformed all other reported LIBs, lithium–air batteries, flow batteries and super-capacitors integrated with a photo-charging component (Supplementary Table 1). These excellent results clearly indicate that the PSCs–LIB developed in this study hold great promise for potential applications as self-chargeable batteries to power the BEVs and various portable electronics.

## Methods

### Materila preparation

The commercial LiFePO_4_ cathode and Li_4_Ti_5_O_12_ anode materials were purchased from the MTI Corporation (USA). The PEDOT:PSS, PbI_2_ and PC61BM were purchased from Clevious, Sigma Aldrich and Nano-C, respectively. CH_3_NH_3_I was synthesized according to the reported procedures^31^.

### PSCs–LIB device assemble

The cathode (anode) was fabricated by blending LiFePO_4_, LFPO (Li_4_Ti_5_O_12_, LTO) with acetylene black carbon and polyvinylidene difluoride at a weight ratio of 90:5:5, respectively. *N*-methyl-2-pyrrolidone was used as the solvent. The slurries were stirred in a sealed glass bottle for 3 h. The resultant LiFePO_4_ and Li_4_Ti_5_O_12_ slurries were then coated on Al and Cu foils, respectively. The LIBs were assembled as the CR 2032 coin-type cells in an Ar-filled glove box. The LFPO–Li and LTO–Li half-cells were assembled from LiFePO_4_ or Li_4_Ti_5_O_12_ with a mass loading of 11–13 mg cm^−2^ as the working electrode, using Li foils as the counter electrode and reference electrode, porous polypropylene film as separator and 1 M LiPF_6_ in a 1:1:1 (v/v/v) mixture of ethylene carbonate, dimethyl carbonate and diethyl carbonate as the electrolyte. The LFPO–LTO full cell was assembled by using LFPO and LTO as the cathode and anode, respectively. The LFPO–Li, LTO–Li and LFPO–LTO cells were measured using an automatic battery tester system (Land, China) as PS and photo-charged or galvanostatically charged and discharged at various current densities in the voltage ranges of 2.5–4.0, 1.0–3.0 and 1.0–2.6 V, respectively (1 C=170 mA g^−1^ for all three samples).

For the PSC construction, ITO glass substrates were cleaned sequentially with detergent, de-ionized water, acetone and iso-propanol, followed by drying with N_2_ flow and ultraviolet–ozone treatment for 15 min. The PEDOT:PSS solution (Al4083 from H. C. Starck) was spin-cast onto ITO electrodes at 5,000 r.p.m. for 40 s. The PEDOT:PSS film was annealed at 140 °C for 10 min to remove residual water. The ITO/PEDOT:PSS substrate was then transferred to evaporator in an Ar-filled glove box for PbI_2_ evaporation (125 nm), followed by dipping the PbI_2_-deposited substrate into a solution of CH_3_NH_3_I in 2-propanol (10 mg ml^−1^) for 40 s to form the CH_3_NH_3_PbI_3_ perovskite layer and being rinsed with 2-propanol for 10 s. Similarly, a second PbI_2_ layer was thermally evaporated onto the pre-formed first layer of CH_3_NH_3_PbI_3_ perovskite film, followed by dipping into the CH_3_NH_3_I solution to form the second layer of CH_3_NH_3_PbI_3_ perovskite, and the process was repeated for three times for a desired thickness. The CH_3_NH_3_PbI_3_ perovskite was then thermally annealed at 100 °C for 10 min in the glove box to complete crystallization for the perovskite film. After the thermal annealing, PC61BM in chlorobenzene solution (17 mg ml^−1^) was deposited onto the perovskite layer by spin coating at 1,000 r.p.m. for 60 s. Finally, the device was transferred to the evaporator for thermal evaporation of Ca (20 nm) and Al (100 nm) at 10^−7^ torr. All the devices were tested in an Ar-filled glove box using a Keithley 2400 source meter and a Newport Oriel sol 2A solar simulator (300 W). We used the 91150V Reference Cell and Meter (ORIEL instrument) to calibrate the light intensity to be 100 mW cm^−2^ before the device testing. The device performance parameters were obtained from the current–voltage curves of the solar cells under illumination. The IPCE was measured on a Solar Cell Measurement System from PV Measurement Inc. The PSCs (single PSC or four single PSCs connected in series) thus fabricted were kept in the glove box under high-purity Ar gas for further electrochemical measurements and long-time rest measurements. The PSCs–LIB units were fabricated by integrating PSCs and a LIB into the system, as shown in Fig. 1.

### Calculations

1. The energy-conversion efficiency of the PSC (*η*_1_):


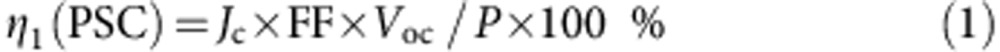


where FF, *V*_oc_, *J*_c_ and P are fill factor, open-circuit voltage (V), short-circuit current density (mA cm^−2^) and incident light power density (100 mW cm^−2^), respectively.

2. The energy-conversion and storage efficiencies for the entire PSCs–LIB integrated unit:


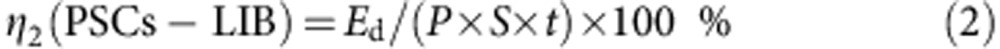


where *E*_d_, *P*, *S* and *t* are discharge energy of LIB (mWh, from Land machine), light power density (100 mWcm^−2^ ), effective area of PSCs in series (cm^−2^) and photo-charge time (h), respectively.

3. The energy storage efficiency for PSC to photo-charge LIB:


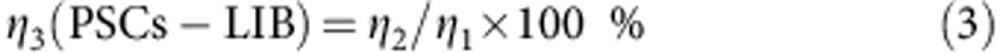


4. The energy storage efficiency for galvanostatically charging LIB with Land machine as power supply (PS-LIB):


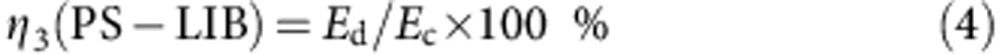


where *E*_d_ and *E*_c_ are the discharge and charge energy (mWh), in which the LIB was galvanostatically charged using Land machine.

## Additional information

**How to cite this article**: Xu, J. *et al.* Efficiently photo-charging lithium-ion battery by perovskite solar cell. *Nat. Commun.* 6:8103 doi: 10.1038/ncomms9103 (2015).

## Supplementary Material

Supplementary Information Supplementary Figures 1-12, Supplementary Tables 1-5, Supplementary Note 1, Supplementary Methods and Supplementary References
