# Supplementary material for: Efficiently photo-charging lithium-ion battery by perovskite solar cell
Source: Nat Commun. 2015 Aug 27;6:8103. doi: 10.1038/ncomms9103 (PMC4560813; doi:10.1038/ncomms9103)
Supplement: Supplementary Information — Supplementary Figures 1-12, Supplementary Tables 1-5, Supplementary Note 1, Supplementary Methods and Supplementary References [file ncomms9103-s1.pdf]

Supplementary Figures

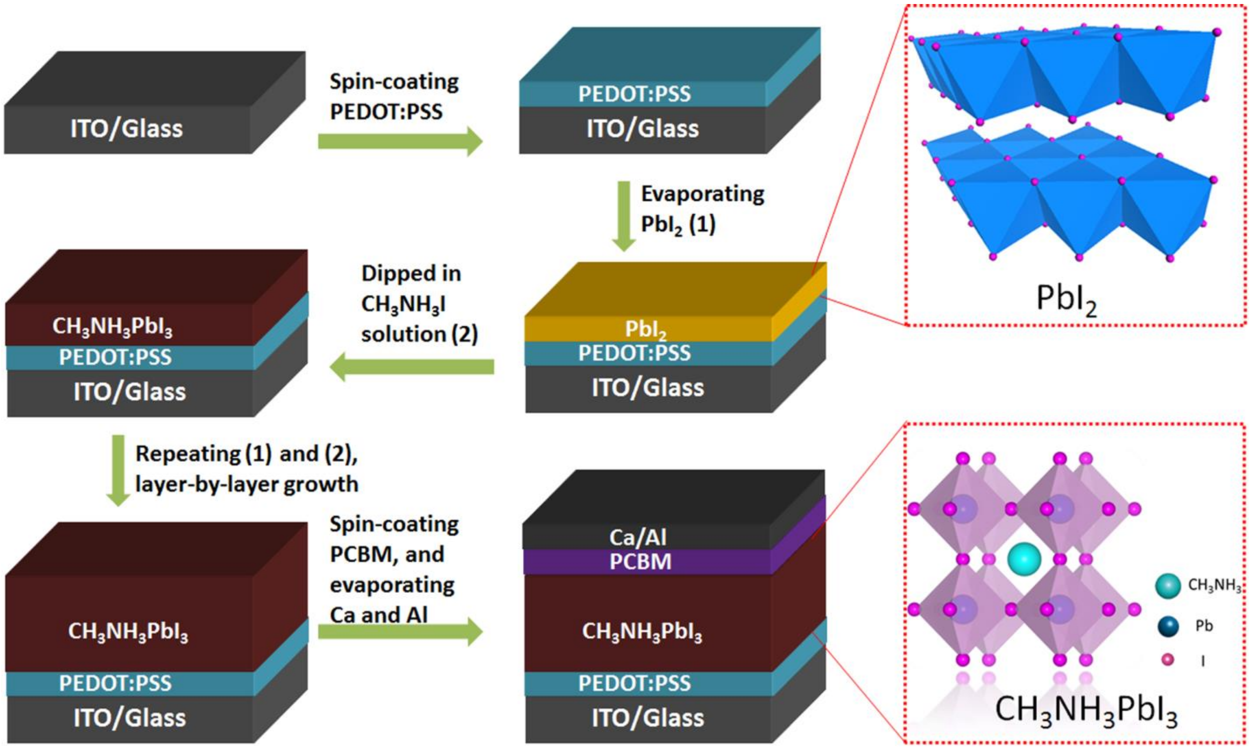

Supplementary Fig. 1. Schematic diagram of the preparation of $CH_3NH_3PbI_3$ perovskite films.The structure and the preparation procedures of $CH_3NH_3PbI_3$ perovskite films.

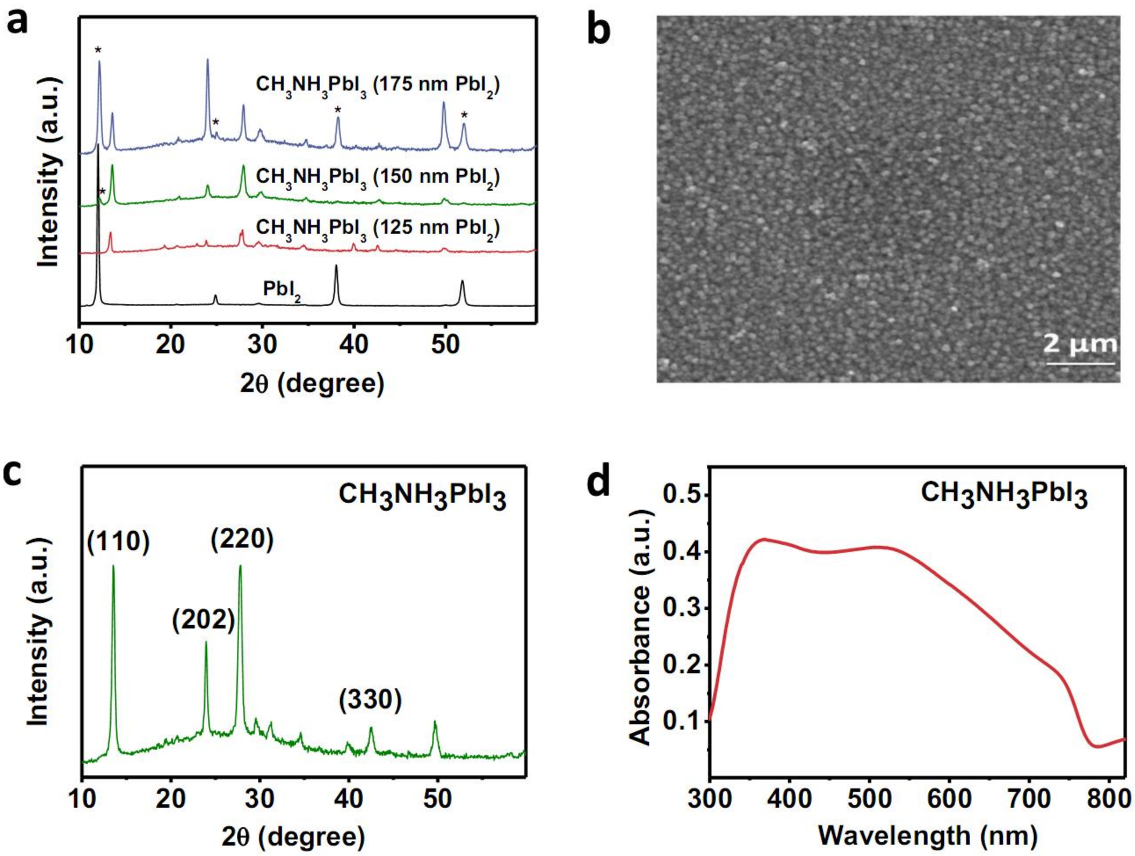

Supplementary Fig. 2. Material characterization of PbI_2 and $CH_3NH_3PbI_3$ perovskite films. (a) XRD patterns of PbI_2 and $CH_3NH_3PbI_3$ perovskite film, (b) SEM image, (c) XRD pattern, and (d) UV-vis absorbance spectra of $CH_3NH_3PbI_3$ film transformed from the dipping of 3-layered PbI_2 with a total thickness of 375 nm in a solution of CH_3NH_3I.

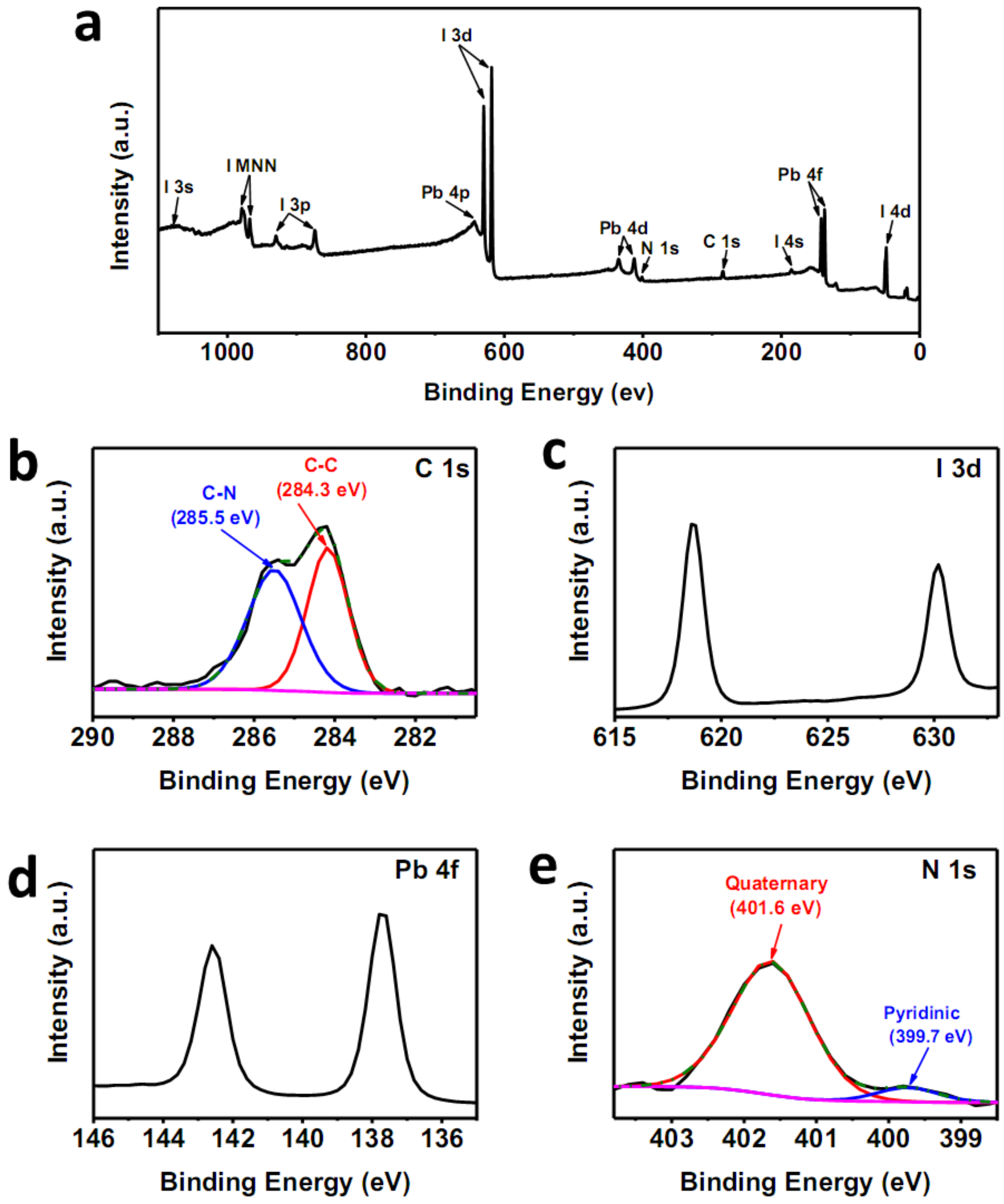

Supplementary Fig. 3. XPS spectra of the $CH_3NH_3PbI_3$ perovskite film. (a) A XPS survey spectrum of $CH_3NH_3PbI_3$ film; and high resolution XPS spectra of (b) C 1s, (c) I 3d, (d) Pb 4f and (e) N1s.

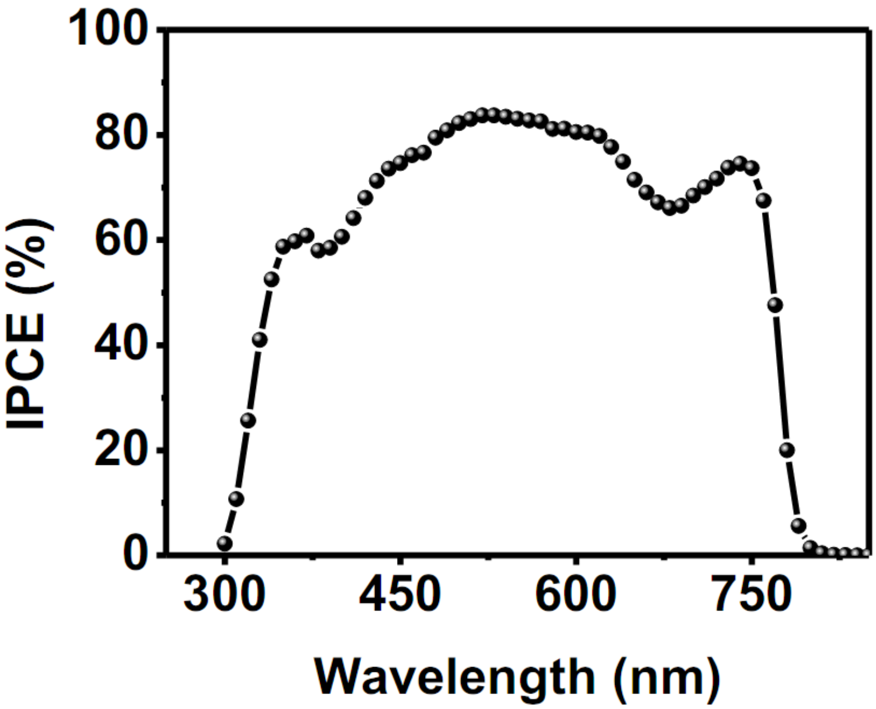

Supplementary Fig. 4. IPCE spectrum of the $CH_3NH_3PbI_3$ perovskite film. The IPCE spectrum of the $CH_3NH_3PbI_3$ perovskite film.

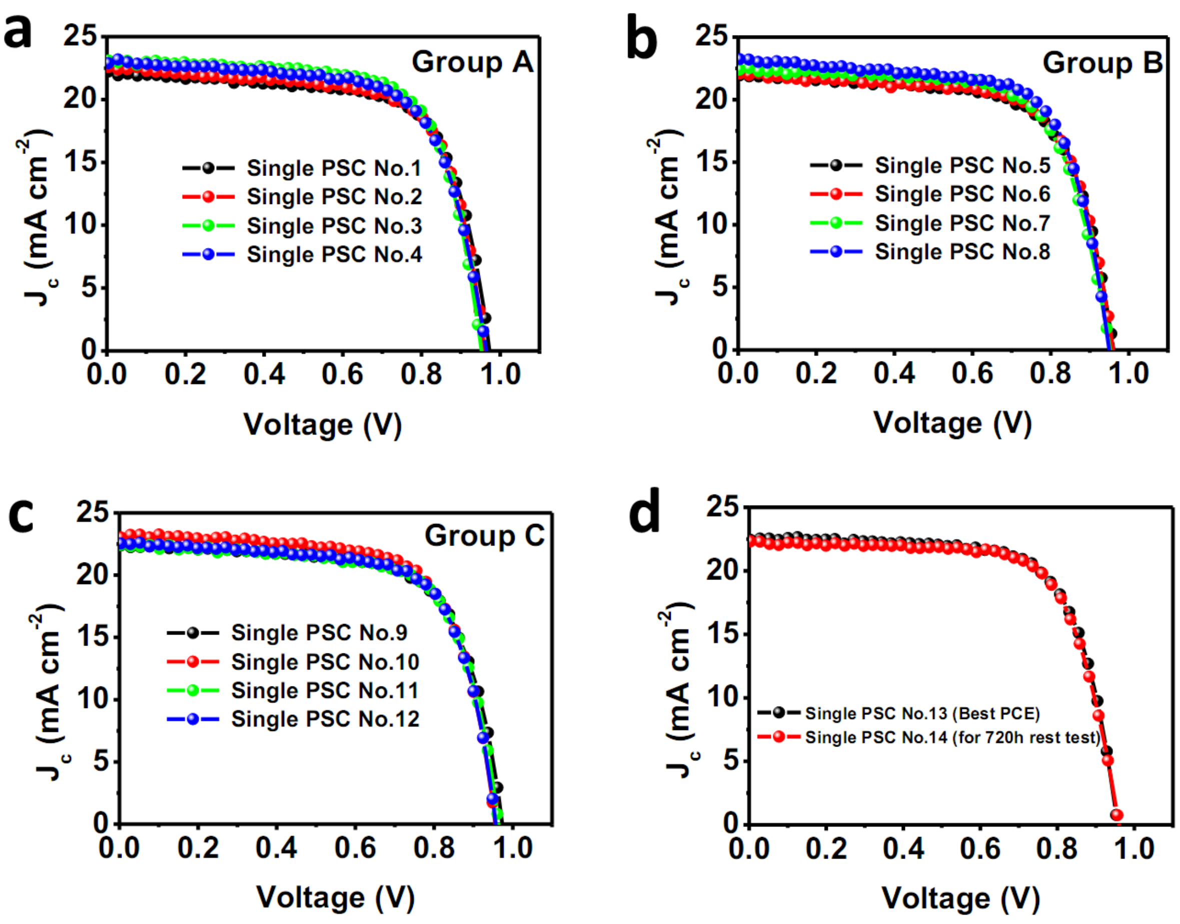

Supplementary Fig. 5. Device performance for $CH_3NH_3PbI_3$ perovskite solar cells (PSCs). J-V curves of randomly selected 14 single PSCs measured under 100 mW cm^{-2} AM 1.5G illumination divided into four groups: (a) four single PSCs (No.1 - No.4, in group A) to be used for fabrication of the fabricated PSCs unit for cycling performance measurements at 0.5 C, (b) four single PSCs (No.5 - No.8, in group B) to be used for fabrication of the fabricated PSCs unit for rate capability measurements at various discharge C-rates, (c) four single PSCs (No.9 - No.12, in group C) to be used for fabrication of the fabricate PSCs unit for long-time rest for 720 hours, (d) single PSC (No. 13, Best) for long-time rest test for 720 hours; and the single PSC with the best η_1 record (single PSC, No. 14, Rest) was also plotted in Supplementary Fig. 5d.

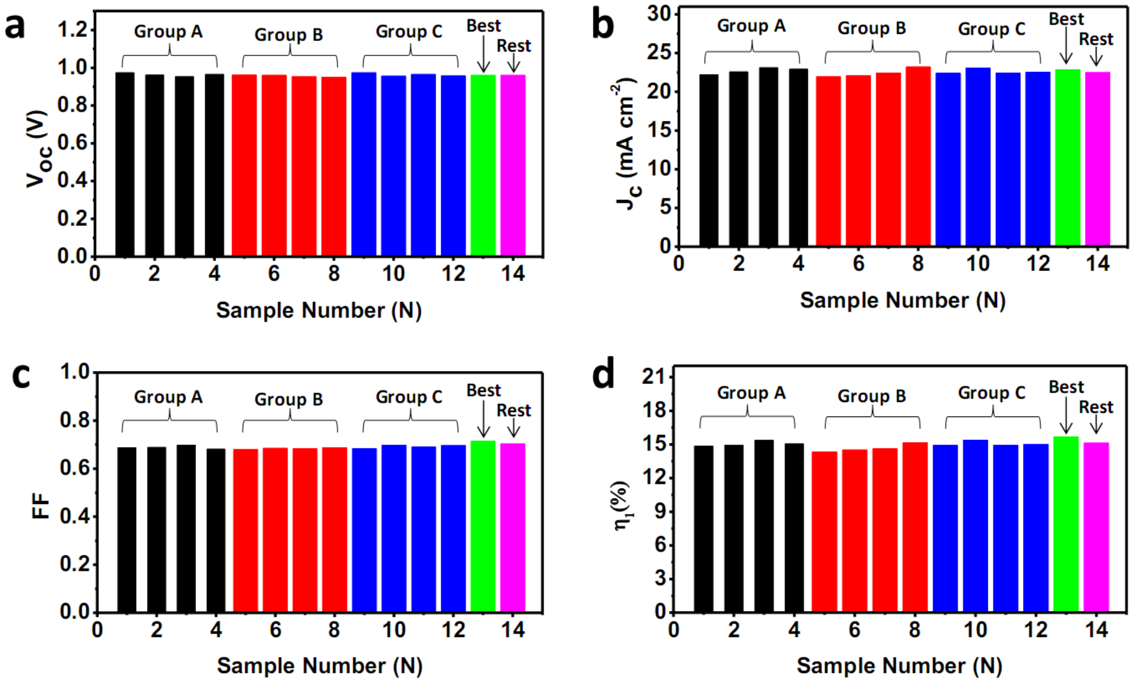

Supplementary Fig. 6. The statistic of key parameters of randomly selected perovskite solar cells (PSCs) based on $CH_3NH_3PbI_3$. (a) V_{oc}, (b) J_c, (c) FF and (d) η_1 of randomly selected 14 single PSCs: four single PSCs (No.1 - No.4, in group A) for the fabrication of PSCs unit for cycling performance measurements at 0.5 C; four single PSCs (No.5 - No.8, in group B) for the fabrication of PSCs unit for rate capability measurements at various discharge C-rates; four single PSCs (No.9 - No.12, in group C) for the fabrication of PSCs unit for long-time rest test for 720 hours; single PSC (No. 13, Best) for long-time rest test for 720 hours; and single PSC (No. 14, Rest) with the best η_1 recorded.

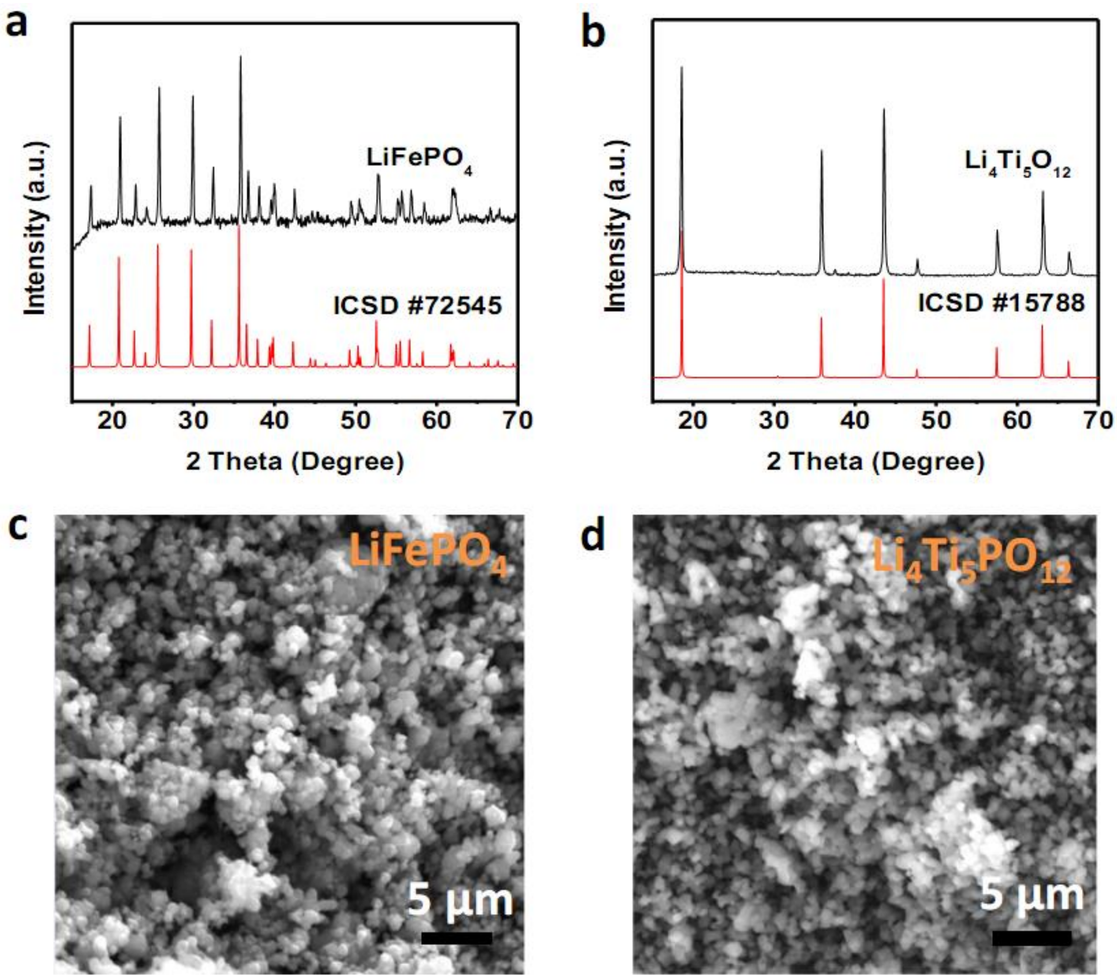

Supplementary Fig. 7. Material characterization of electrode materials for LIB. XRD patterns of (a) $LiFePO_4$ and (b) $Li_4Ti_5O_{12}$, and SEM images of (c) $LiFePO_4$ and (d) $Li_4Ti_5O_{12}$.

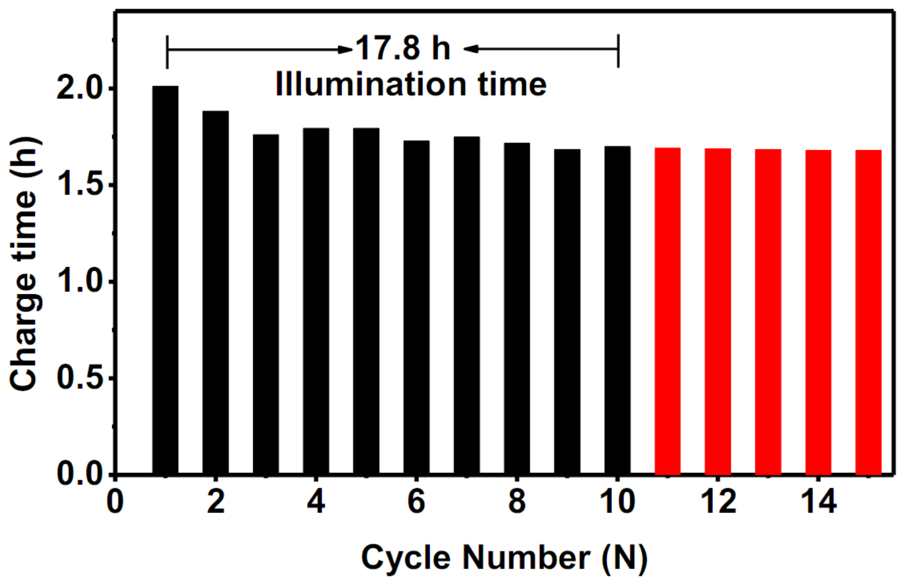

Supplementary Fig. 8. Comparison of photo-charge and galvanostatic charge time of the devices at the same C-rate. Photo-charged time at an photocharge current density of 0.5 C (*Black*, PSCs-LIB) and galvanostatic charged time at 0.5 C (*Red*, PS-LIB) *vs.* cycle number.

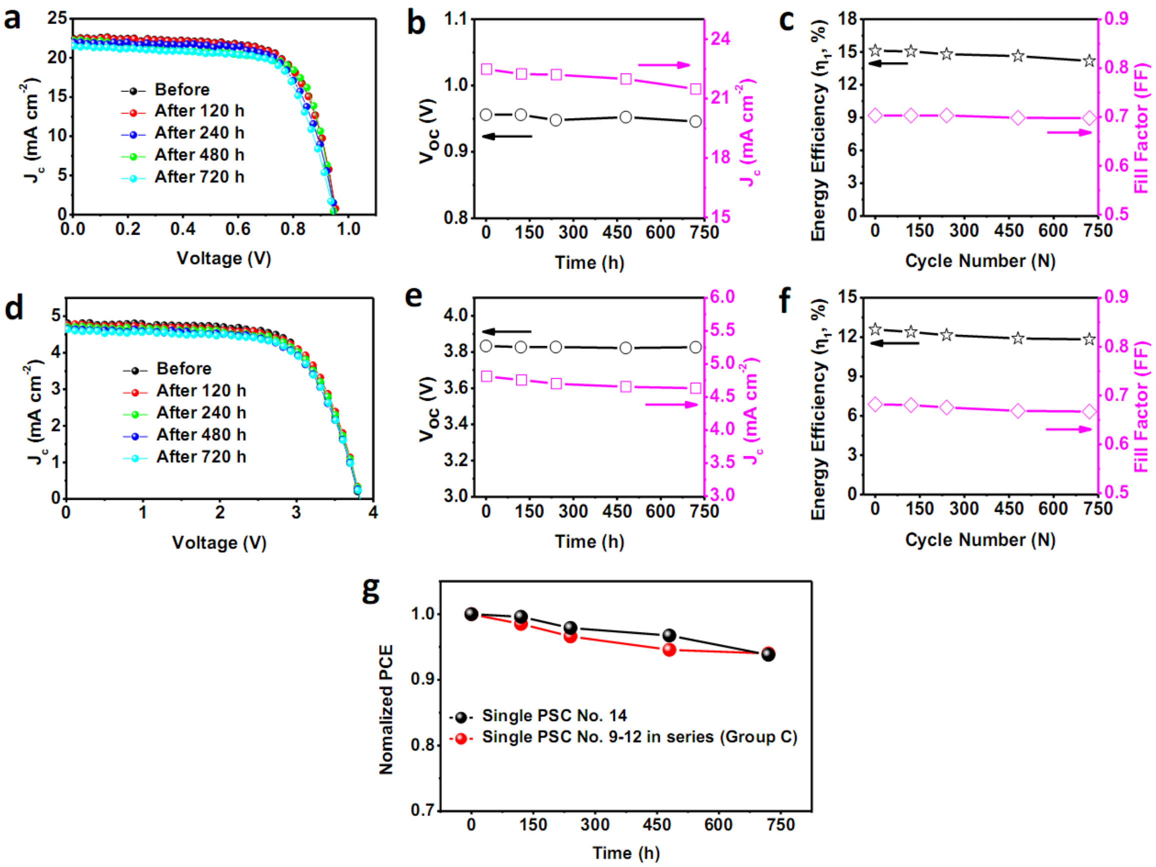

Supplementary Fig. 9. Device performance of single PSC and fabricated PSCs unit after long time rest. (a) J-V curves, (b) V_{oc} and Jc, (c) FF and η_1 of a single PSC (No. 14); (d) J-V curves, (e) V_{oc} and J_c, (f) FF and η_1 of the fabricated PSCs unit (fabrication from four single PSCs (No. 9- NO.12 in group C)) after various hours. (g) PCE (η_1) decay of the devices measured based on the single PSC/fabricated PSCs unit unencapsulated and stored in a high-purity Ar-filled glove box for 720 hours.

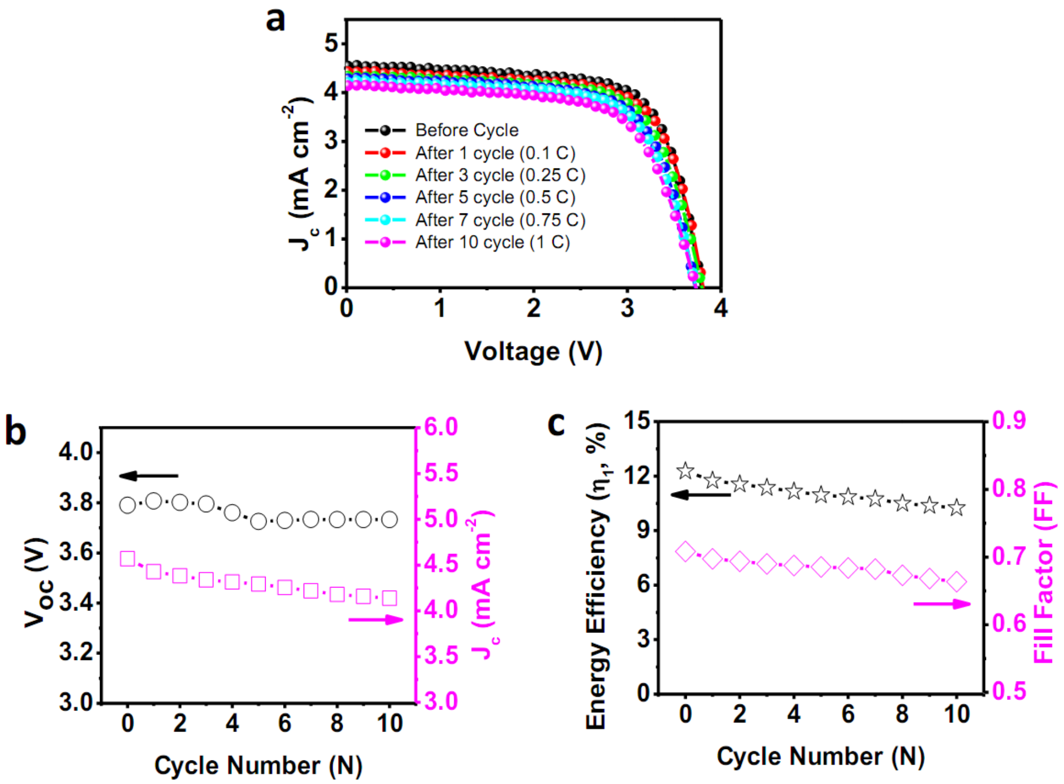

Supplementary Fig. 10. Performance of connected PSCs device after the rate capability measurements with LFPO-LTO cell (four single PSCs (No. 9- NO.12, in group C) in the PSCs-LIB unit). (a) J-V curves of fabricated PSCs before and after different cycles, (b) V_{oc} and J_c and (c) fill factor (FF) and solar-to-electric power conversion efficiency (PCE, η_1, Method calculation 1).

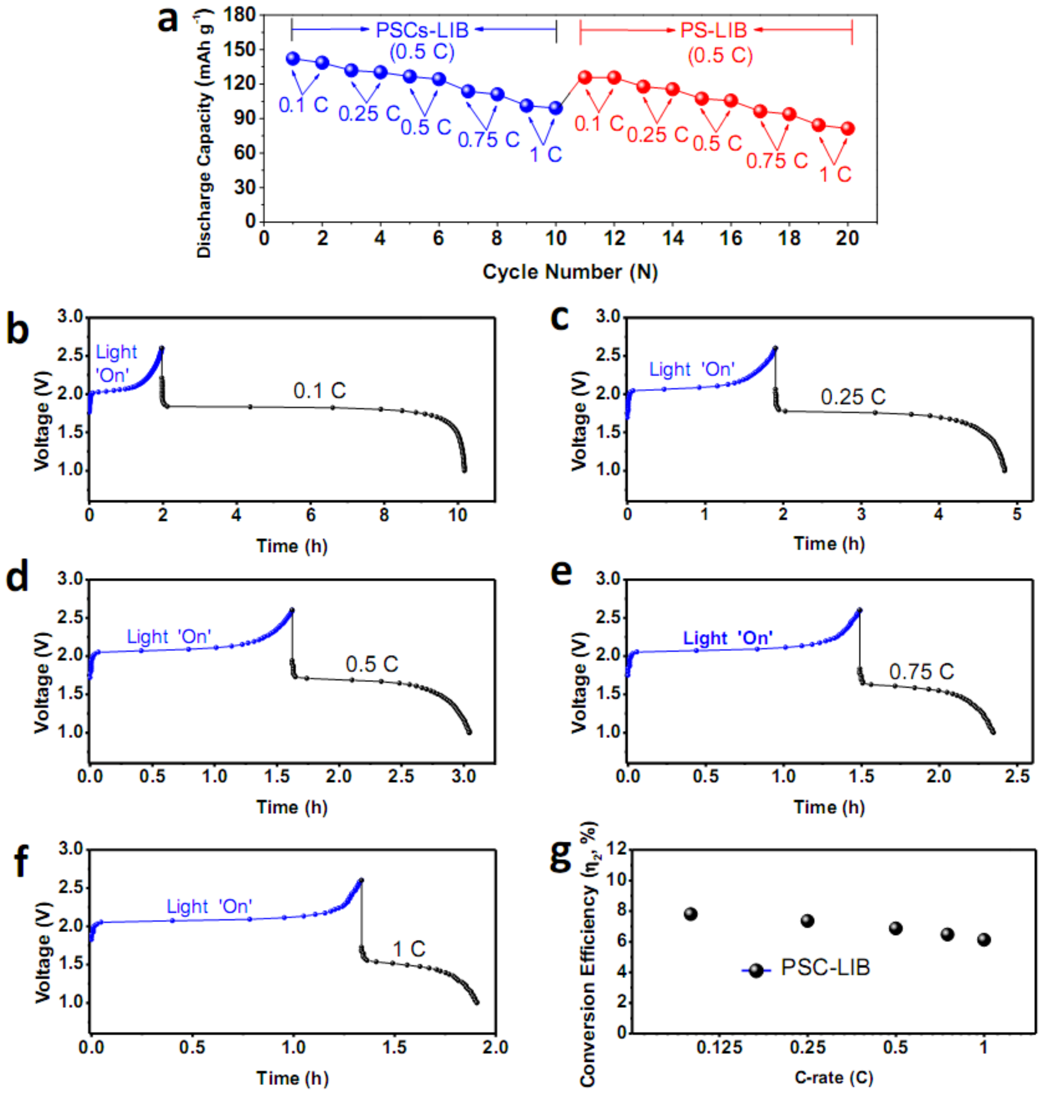

Supplementary Fig. 11. Electrochemical performance and energy conversion efficiency of PSCs-LIB and PS-LIB. (a) Discharge capacity of LFPO-LTO cell at various C-rates (*Blue line* in the 1-10th cycles: photo-charge at 0.5 C using PC and galvanostatically discharge at various C-rates using PS machine. *Red line* in the 11th-20th cycles: galvanostatically charged at 0.5 C and galvanostatically discharged at various C-rates using Land machine). Voltage-time (V-t) curves of photo-charged PSCs-LIB unit, followed by galvanostatically discharging at (b) 0.1 C, (c) 0.25 C. (d) 0.5 C. (e) 0.75 C (f) 1 C. (g) overall photo-electric conversion and storage efficiency of the entire PSCs-LIB unit (η_2) *vs.* C-rates.

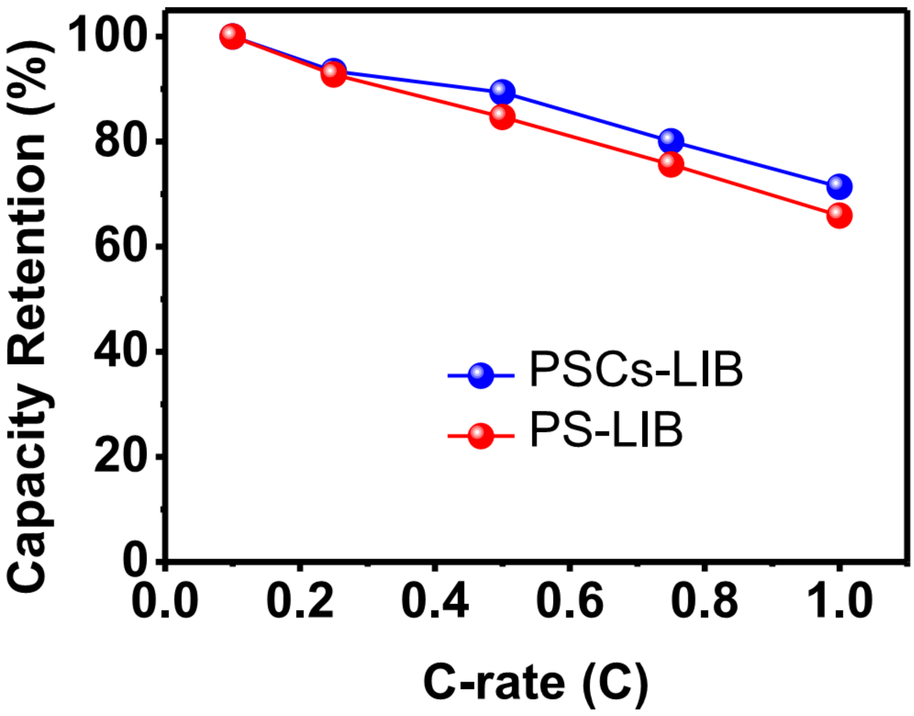

Supplementary Fig. 12. Discharge rate capability comparison between PSCs-LIB and PS-LIB. Capacity retention *vs.* C-rates of PSCs-LIB and PS-LIB.

Supplementary Tables

Supplementary Table 1. Reporetd integrated devices of solar-storage/conversion systems.

Integrated device	η_2* (%)	η_1* (%)	Additional information	Supplementary Ref.
Solar batteries	-	-	This integrated device was composed of two electrodes with intrinsic Si particles bound with ferroelectric PVDF particles, a $LiPF_6$ in a solution of EC:DMC (volume ratio 1:1), and a PVDF membrane. The device can be photo-charged under 1000 W m^{-2} of white light to an open-circuit voltage of 0.47 V with a capacity of 37.62 mC cm^{-2}.	2
Photo-LIB	0.82	-	The solar cell part was made of two different connected solar cells based on TiO_2 nanorod arrays and nanotubles, respectively, which provided an open-circuit voltage of 3.39 V and a short-circuit current density of 1.01 mA cm^{-2}. The power pack was solar-illumination charged and galvanostatically discharged between 0.75 and 3 V on a program-controlled test system. The power pack can be charged to about 3 V in about 8 min, and the discharge capacity is about 38.89 μAh under the discharge density of 100 μA.	3
A solar rechargeable battery based on polymeric charge storage electrode	-	-	A solar rechargeable battery was constructed by using a hybrid TiO_2/poly(3,4-ethylenedioxythiophene, PEDOT) photo-anode and a ClO_4^- doped polypyrrole counter electrode. A potential voltage range of 0.25-0.76 V of the cell at photo-charge under irradiation and electrochemical discharge at 8 μA cm^{-2} were demonstrated. The discharge capacity calculated from the amount of the loaded PEDOT on the hybrid photo-anode was about 8.3 mAh g^{-1}.	4
solar rechargeable battery	-	-	When the cutoff potentials for charge and discharge were set in the voltage range of 0.2 - 1.1 V, the device showed the discharge capacity of 0.200 mAh cm^{-2} in the first cycle.	5
Photo-capacitor	2.1	4.56	The integrated device was fabricated by a fiber and PANi-based DSSC. The photoelectric conversion efficiency of the device can reach up to 5.41%. The device delivered an area-specific capacitance of 3 mF cm^{-2} to 41 mF cm^{-2}.	6
Solar rechargeable flow battery	-	-	This integrated battery was fabricated by a dye-sensitized solar cell (DSSC) and redox flow batteries (RFBs). The maximum discharge capacity obtained from the RFB was 53.3 mAhL^{-1}, corresponding to a 47.9% utilization of the photocharge	7

			capacity.	
Solar rechargeable flow battery	-	-	The solar rechargeable redox flow battery was fabricated with dye-sensitized TiO_2 as photo-anode, soluble Li_2WO_4 as anode-active species in aqueous electrolyte, LiI as cathode-active species in organic electrolyte, and LISICON lithium-ion-conducting glass ceramic film as membrane. The initial discharge capacity of the battery was 0.0153 mA h mL^{-1}, in the voltage range of 0.2 -0.76 V. A discharge capacity of 0.0151 mA h mL^{-1} was retained after 10 cycles.	8
Dye-sensitized solar cell with energy storage	-	3.7	The device was fabricated by a photoelectrode of dye-sensitized TiO_2 nanoparticles and a PVDF/ZNWA nanocomposite modified counter electrodes (CEs), The CEs of DSSCs were modified by PVDF/ZnO nanowire array nanocomposites for the energy storage function. The device with 25-nm-thick PVDF could provide a η of 3.70% while part of the photo-generated charge was stored in CE with a charge density of 2.14 C g^{-1}	9
Photo-capacitor	0.79	2.31	The MWCNT–PANI composite as DSSC counter electrode showed a V_{OC} of 0.75 V, J_c of 5.10 mA cm^{-2}, and FF of 0.61, which produced a η of 2.31%. The flexible integrated device was photocharged to 0.73 V in 183 s and then maintained. The galvanostatic discharge time was about 137 s at a discharge current density of 1.4 mA cm^{-2}. The specific capacitance was 83 F g^{-1}, and the energy storage efficiency was calculated to be 34% with an entire energy conversion and storage efficiency of 0.79%.	10
Photo-capacitor	-	2.73	Dye-sensitized solar cell and electrochemical capacitor were coaxially integrated into a novel “energy fiber”. The maximum photoelectric conversion efficiency achieved was 2.73%, while the energy storage efficiency reached 75.7% with specific capacitances up to 0.156 mF cm^{-1} or 3.32 mF cm^{-2} and power densities up to 0.013 mW cm^{-1} or 0.27 mW cm^{-2}.	11
Photo-capacitor	-	< 6-7	The as-received commercial polycrystalline silicon solar cell panel (Silicon Solar, 400 mA, 22 W, Polycrystalline, 14.8% efficiency) was adopted as the photo-charging source. The device was operated ideally to present that the supercapacitor, 84% of the charge generated by the PV device can be effectively stored by the supercapacitor.	12
Photo-supercapacitor	1.64	-	This integrated device was fabricated based on bi-polar ATO Bi-polar TiO_2 nanotube arrays with plasma treatment. The cycle stability of the photo-supercapacitor was investigated for 100 cycles at a discharge current density of 0.1 $mAcm^{-2}$. The energy density of the device was calculated to be 6.662×10^{-8} Wh cm^{-2}. The device exhibited a maximum energy storage efficiency of	13

			51.60% and an overall photoelectric conversion and storage efficiency of 1.64%.	
Photo-lithium oxygen battery	-	-	This device was fabricated by integrating a redox-coupled dye-sensitized photo-electrode into a lithium oxygen battery for photo-assisted charging. Due to the contribution of the photo-voltage, the charging over-potential is reduced from ~ 3.8 to ~ 3.40 V.	14
Photo-capacitor	0.79	-	Integrated polymer solar cell and electrochemical supercapacitor in a flexible and stable fiber format was fabricated. An entire photoelectric conversion and storage efficiency of 0.79% before bending, which was decreased significantly to 0.05% after bending for 100 cycles.	15
Photo-capacitor	2.1	3.2	An integrated wearable energy device based on aligned CNT/PANI composite fiber was fabricated. The energy storage efficiency of the integrated energy textile was calculated to be 68.4% for the CNT/PANI composite. The entire energy conversion efficiency was 2.1% for the integrate device.	16
Photo-capacitor	-	-	This device was made of poly ethylenedioxythiophene:polystyrene sulfonate and (PEDOT:PSS) and a porphyrin dye, which showed a capacitance of ~1.04 mF. The device was charged up to 430 mV (open circuit voltage) under a solar simulated illumination and stored the charge for more than 10 min in the dark.	17
Photo-capacitor	-	12.6	This device was fabricated by a $CH_3NH_3PbI_3$ solar cell and a polypyrrole super-capacitor. A max energy storage efficiency of 10% can be achieved when the voltage of the device was charged to 0.7 V for 300 s.	18
Photo-capacitor	1.83	2.66	This device was fabricated from solar cells based on CdS/CdSe quantum dots (QDs) co-sensitized with carbon. The device was photo-charged and galvanostatic discharged at 20 μA cm^{-2} in a low voltage range of < 0.24 V, with an energy storage of 56.4 μJ.	19
Photo-LIB	7.80	12.65	This fabricated unit consisting of four perovskite ($CH_3NH_3PbI_3$) solar cells (PSCs) connected in series with an outstanding solar-to-electric power conversion efficiency (PCE) of 12.65%. The fabricated PSCs-LIB device demonstrated a high photo-electric conversion storage efficiency of 7.36% at 0.5 C and excellent cycling stability with a long illumination period of 17.8 hours. At a discharge C-rate of 0.1 C, the PSCs-LIB exhibited even a higher photo-electric conversion storage efficiency of 7.80%.	This Work

* η_1: Power conversion Efficiency of solar cell. η_2: Overall photo-electric conversion efficiency of the integrate device.

Supplementary Table 2. XPS results of the $CH_3NH_3PbI_3$ film.

**Atomic element**	**C**	**Pb**	**I**	**N**
**Percentage (at. %)**	41.6	12.8	36.5	9.1

Supplementary Table 3. Performance parameters of the $CH_3NH_3PbI_3$ perovskite solar cells for photo-charging LIBs at 0.5 C for 10 cycles.

**Cycle number (N)**	**J_c (mA cm_{-2})**	**V_{oC} (V)**	**FF**	**PCE-PSCs (η_1, %)**	**Average PCE* (η_1, %)**
Before cycle	4.83	3.85	0.68	12.65	N/A
1	4.75	3.84	0.68	12.40	12.52
2	4.69	3.84	0.68	12.24	12.32
3	4.64	3.83	0.68	12.08	12.16
4	4.59	3.84	0.68	12.00	12.04
5	4.52	3.82	0.69	11.91	11.95
6	4.46	3.82	0.68	11.60	11.76
7	4.38	3.80	0.68	11.31	11.45
8	4.38	3.83	0.67	11.23	11.27
9	4.37	3.81	0.67	11.20	11.21
10	4.37	3.81	0.67	11.16	11.18

* In order to calculate energy conversion of LIB ($\eta_3 = \eta_2/\eta_1$, see calculation in Section 4 of SI) at each cycle, the power conversion efficiency of PSCs (PCE-PSCs, η_1) for LIB in the whole charge process at n^{th} cycle was assumed to be an average PCE, which was calcualted by the (PCE (n^{th-1}) + PCE (n^{th}))/2. For example, average PCE at 1st cycle = (12.65%+12.40%)/2=12.52%.

Supplementary Table 4. Photo-electric conversion and storage effciency of the entired fabricated PSCs-LIB unit (η_2, %, PCE-PSCs-LIB) at each cycle with a galvanostatic discharge rate of 0.5 C and its corresponding power storage efficiency of LIB (η_3, PCE-LIB).

**Cycle number (N)**	**PCE-PSCs-LIB (η_2, %)**	**PCE-LIB (η_3,%)**
1	7.08	56.57
2	7.31	59.40
3	7.36	60.62
4	6.92	57.66
5	6.92	58.17
6	6.98	59.64
7	6.74	57.20
8	6.76	60.12
9	6.73	60.08
10	6.82	61.09
11	-	66.25**
12	-	66.32**
13	-	66.29**
14	-	65.97**
15	-	66.02**

** The effiency of PS-LIB was calculated based on Method calculation 4**.**

Supplementary Table 5. Photo-electric conversion and storage effciency of the entirr fabricated PSCs-LIB device (η_2, **%,** PCE-PSCs-LIB) at different discharge C-rates.

**C-rates (C)**	**PCE-PSCs-LIB (η_2, %)**
0.1	7.80
0.25	7.35
0.5	6.87
0.75	6.47
1	6.11

Supplementary Note

To demonstrate repeated fabrication of perovskite SCs and fabricated PSCs units without large statistic deviations for reliably photo-charging LIBs, we have prepared more new perovskite SCs and connected PSCs units for testing. Supplementary Figs. 5, 6 and 11 show reproducible and stable photovoltaic parameters, including J_c, V_{oc}, FF, and η_1, for all the single PSC and their connected units tested in this study. In order to determine the discharge rate capability of LFPO-LTO cell in the PSCs-LIB system, we photo-charged the LFPO-LTO LIB with the perovskite SC at 0.5 C and galvanostatically discharged at various C-rates from 0.1 to 1 C (*black dots*, Supplementary Fig. 11a). Supplementary Figs. 11b-f show typical charge-discharge behaviors of the photo-charged LFPO-LTO cell discharged at different C-rates, which agreed well with the results shown in Figure 2f. As can be seen, the PSCs-LIB cell delivered reversible capacities of 142.1, 131.9, 126.6, 113.7 and 101.2 mA h g^{-1} at discharge current densities of 0.1, 0.25, 0.5, 0.75, 1 C, respectively. As expected, the initial capacity of the PS-LIB cell at 0.1 C is very close to the initial reversible capacity of 140.4 mAh g^{-1} for the cell at 0.5 C (Figure 3e). Supplementary Fig. 11a (*red dots*) also shows the discharge rate capability at various C-rates from 0.1 to 1 C after galvanostatically charged at 0.5 C for the LFPO-LTO cell, which has been photo-charged and galvanostatically charged for 10 cycles (*i.e.*, blue dots in Supplementary Fig. 11a). As can be seen in Supplementary Fig. 12, the photo-charged PSCs-LIB and the PS-LIB cell gavanostatically charged and discharged at 0.5 C both exhibited a similar rate capability from 0.1 - 1 C, further indicating a highly stable PSCs-LIB system.

Supplementary Methods

Materials preparation

The commercial $LiFePO_4$ cathode and $Li_4Ti_5O_{12}$ anode were purchased from the MTI Corporation (USA). The PEDOT:PSS, PbI_2, and PC61BM were purchased from Clevious, Sigma Aldrich, and Nano-C, respectively. CH_3NH_3I was synthesized according to the reported procedures (Supplementary Ref. 1).

Materials characterization

Scanning electron microscopic (SEM) images were recorded on a Nova nano SEM 600. X-ray diffraction (XRD) was conducted on a Miniflex II Desktop X-ray diffractometer. UV-vis absorption was measured on a Shimadzu UV1800 spectrometer. The incident photon-to-current efficiency (IPCE) was measured on a Solar Cell Measurement System from PV measure ment Inc.

PSCs-LIB device assemble

The cathode (anode) was fabricated by blending $LiFePO_4$,LFPO, ($Li_4Ti_5O_{12}$, LTO) with acetylene black carbon and PVDF, at a weight ratio of 90 : 5 : 5, respectively. N-methyl-2-pyrrolidone (NMP) was used as the blending solvent for the mixture. The slurries were stirred in a sealed glass bottle for 3 hours. The obtained $LiFePO_4$ and $Li_4Ti_5O_{12}$ slurries were coated on Al and Cu foils, respectively, and then dried at 90 °C for 12 h. Thereafter, the electrodes were vacuum-dried again at 90 °C for 12 h after having been punched into round disks (2 cm^{-2}) with a

mass loading of 22 ~ 26 mg. The LIBs were then assembled using CR 2032 coin-type cells in an Ar-filled glove box. The LFPO-Li and LTO-Li half cells were assembled using $LiFePO_4$ or $Li_4Ti_5O_{12}$ as the working electrode, Li foil as the counter electrode and reference electrode, porous polypropylene film as separator and 1 M $LiPF_6$ in a 1:1:1 (v/v/v) mixture of ethylene carbonate (EC), dimethyl carbonate (DMC) and diethyl carbonate (DEC) as the electrolyte. The LFPO-LTO full cell was assembled by using LFPO and LTO as the cathode and anode, respectively. The LFPO-Li, LTO-Li, and LFPO-LTO cells were measured using an automatic battery tester system (Land®, China) and photo-charged or galvanostatically charged and discharged at various current densities in the voltage ranges of 2.5 - 4.0, 1.0 - 3.0 and 1.0 - 2.6 V, respectively (1 C = 170 mA g^{-1} for all three samples).

ITO glass substrates were cleaned sequentially with detergent, de-ionized water, acetone, and iso-propanol, followed by drying with N_2 flow and UV-ozone treatment for 15 minutes. The PEDOT:PSS solution (Al4083 from H. C. Starck) was spin-cast onto ITO electrodes at 5000 rpm for 40 s, followed by heating at 140 °C for 10 minutes. The PEDOT:PSS-coated ITO/Glass substrate was then transferred to an evaporator in an Ar-filled glovebox for PbI_2 evaporation (125 nm). Thereafter, the PbI_2-deposited substrate was dipped into a solution of CH_3NH_3I in 2-propanol (10 mg ml^{-1}) for 40 s to form the $CH_3NH_3PbI_3$ perovskite layer and rinsed with 2-propanol. Similarly, a second PbI_2 layer was thermally evaporated onto the $CH_3NH_3PbI_3$ perovskite film, followed by dipping into the CH_3NH_3I solution to form the second layer of $CH_3NH_3PbI_3$ perovskite, and the process repeated for 3 times for a desired thickness was obtained. The $CH_3NH_3PbI_3$ perovskite thus formed was then thermally annealed at 100 °C for 10 minutes in the glove box to complete crystallization of the perovskite and to eliminate the interface, if any, through the film thickness. After the annealing, PC61BM in chlorobenzene

solution (17 mg ml^{-1}) was deposited onto the perovskite layer by spin-coating at 1000 rpm for 60 s. Finally, the device was transferred to the evaporator for thermal evaporation of Ca (20 nm) and Al (100 nm) at 10^{-7} Torr. The fabricted solar cell was kept in the glove box under high purity Ar gas and ready for further electrochemical measurements and long-time rest measurements.

Calculations

The energy-conversion efficiency of the peroveskite solar cell (PSC)(η_1):

$$\eta_1(PSC) = J_c \times FF \times V_{OC} / P \times 100\ \% \qquad (1)$$

where FF , V_{OC} , Jc , and P are fill factor, open-circuit voltage (V),short-circuit current density (mA cm^{-2}), and incident light power density (100 mWcm^{-2}), respectively.

The energy conversion and storage efficiency of the entire perovskite solar cells (PSCs) - lithium ion battery (LIB) (PSCs-LIB) fabricated unit

$$\eta_2\ (PSCs\text{-}LIB) = E_d / (P \times S \times t) \times 100\ \% \qquad (2)$$

where E_d, P, S and t are discharge energy of LIB (mWh, from Land machine), light power density (100 mWcm^{-2}), effective area of PSCs in series (cm^{-2}) and photo-charge time (h), respectively.

The energy storage efficiency for perovskite solar cells (PSCs) to photo-charge lithium ion battery (LIB) :

$$\eta_3\ (PSCs\text{-}LIB) = \eta_2 / \eta_1 \times 100\ \% \qquad (3)$$

The energy storage efficiency for galvanostatically charging lithium ion battery with Land machine (PS-LIB)

$$\eta_3 \text{ (PS-LIB)} = E_d/E_c \times 100\ \% \quad (4)$$

where E_d (Wh) and E_c (Wh) are the discharge and charge energy (mWh) in which the LIB was galvanostatically charged using Land machine as power supply (PS).

Supplementary References

1. Xiao, Z. et al. Giant switchable photovoltaic effect in organometal trihalide perovskite devices. *Nat. Mater*. **14**, 193-198 (2015).

2. Lo, C.-W., Li, C. & Jiang, H. Direct solar energy conversion and storage through coupling between photoelectrochemical and ferroelectric effects. *AIP Adv*. **1**, 042104 (2011).

3. Guo, W., Xue, X., Wang, S., Lin, C. & Wang, Z.L. An integrated power pack of dye-sensitized solar cell and Li battery based on double-sided TiO_2 nanotube arrays. *Nano Lett*. **12**, 2520-2523 (2012).

4. Liu, P., Yang, H.X., Ai, X.P., Li, G.R. & Gao, X.P. A solar rechargeable battery based on polymeric charge storage electrodes. *Electrochem. Commun.* **16**, 69-72 (2012).

5. Yan, N.F., Li, G.R., Pan, G.L. & Gao, X.P. TiN nanotube arrays as electrocatalytic electrode for solar storable rechargeable battery. *J. Electrochem. Soc.* **159**, A1770-A1774 (2012).

6. Fu, Y. et al. Integrated power fiber for energy conversion and storage. *Energ. Environ. Sci.* **6**, 805-812 (2013).

7. Liu, P. et al. A solar rechargeable flow battery based on photoregeneration of two soluble redox couples. *ChemSusChem* **6**, 802-806 (2013).

8. Yan, N.F., Li, G.R. & Gao, X.P. Solar rechargeable redox flow battery based on Li2WO4/LiI couples in dual-phase electrolytes. *J. Mater. Chem. A* **1**, 7012-7015 (2013).

9. Zhang, X., Huang, X., Li, C. & Jiang, H. Dye-sensitized solar cell with energy storage function through PVDF/ZnO nanocomposite counter electrode. *Adv. Mater.* **25**, 4093-4096 (2013).

10. Yang, Z. et al. An integrated device for both photoelectric conversion and energy storage based on free-standing and aligned carbon nanotube film. *J. Mater. Chem. A* **1**, 954-958 (2013).

11. Chen, X. et al. A novel "energy fiber" by coaxially integrating dye-sensitized solar cell and electrochemical capacitor. *J. Mater. Chem. A* **2**, 1897-1902 (2014).

12. Westover, A.S. et al. Direct integration of a supercapacitor into the backside of a silicon photovoltaic device. *Appl. Phys. Lett.* **104**, 213905 (2014).

13. Xu, J. et al. Integrated photo-supercapacitor based on Bi-polar TiO_2 nanotube arrays with selective one-side plasma-assisted hydrogenation. *Adv. Func. Mater.* **24**, 1840-1846 (2014).

14. Yu, M., Ren, X., Ma, L. & Wu, Y. Integrating a redox-coupled dye-sensitized photoelectrode into a lithium–oxygen battery for photoassisted charging. *Nature Commun.* **5**, 5111, (2014).

15. Zhang, Z. et al. Integrated polymer solar cell and electrochemical supercapacitor in a flexible and stable fiber format. *Adv. Mater.* **26**, 466-470 (2014).

16. Pan, S. et al. Wearable solar cells by stacking textile electrodes. *Angew. Chem. Int. Ed.* **24**, 6224-6228. (2014).

17. Takshi, A., Yaghoubi, H., Tevi, T. & Bakhshi, S. Photoactive supercapacitors for solar energy harvesting and storage. *J. Power Sources* **275**, 621-626 (2015).

18. Xu, X. et al. A power pack based on organometallic perovskite solar cell and supercapacitor. *ACS Nano* **9**, 1782-1787 (2015).

19. Shi, C. et al. An "all-in-one" mesh-typed integrated energy unit for both photoelectric conversion and energy storage in uniform electrochemical system. *Nano Energy*. doi:10.1016/j.nanoen.2015.03.032 (2015).
